# Analysis on 1481 case of medical complaints in a Tertiary Hospital in Fujian Province: A 5-year retrospective study

**DOI:** 10.1097/MD.0000000000034107

**Published:** 2023-06-30

**Authors:** Jian Jiang, Fanqian Huang, Huiting Li

**Affiliations:** a The School of Public Health, Fujian Medical University, Fuzhou, China; b Fujian Provincial Hospital, Fuzhou, China.

**Keywords:** hospital, medical complaint, patient experience, patient safety, patient satisfaction, patients’ complaints, patients’ praise

## Abstract

This study aims to review the 1481 cases of medical complaints from Fujian Provincial Jinshan Hospital in the past 5 years for providing a reference for new hospitals to deal with medical complaints, optimize medical procedures, improve medical quality, and enhance patient experience. The medical complaint information received by the hospital’s medical department and service center accepted and transferred by the health administrative department in the past 5 years was systematically reviewed and statistically analyzed by using hierarchical clustering method. The transfer of the health administration department (61.5%) and the acceptance of the service center (28.9%) were the main sources of medical complaints in hospital. The incidence of medical complaints per 10,000 patients in the hospital was between 3 and 6. The maximum number of complaints was 2017 (5.28 cases/10,000 population), and the least was in 2019 (3.2 cases/10,000 population). The median of complaints was 25, and May to Sep was the period of high incidence of medical complaints each year. In 5 years, the month with the largest number of complaints was May 2020 (41 cases), followed by August 2017(40 cases), and the month with the least number was November 2020 (11 cases). In the past 5 years, the hospital’s medical complaints were mainly in 4 aspects: medical process (n = 329, 22.2%), medical environment (n = 282, 19%), humanistic care (n = 277, 18.7%), and medical management (n = 209, 14.1%). The most frequent complaints were in clinical departments, among which the emergency, outpatient, and pediatric departments accounted for more than 50%. The top 3 complaints were doctors (n = 778, 53%), logistics (n = 284, 19%), and nurses (n = 239, 16%). The main way to resolve complaints was letter and telephone feedback (n = 1372, 92.6%). Our research recommends that new hospitals change their concepts, pay more attention to the services and quality of medical resources and logistical support, follow the best practices of patient-centered, perfect various medical complaint channels, and establish multiple methods. They should also properly accept and dispose medical complaints, improve the timeliness and feedback efficiency of responding to medical complaints, strengthen communication, exchange, and dialogue, and improve patients’ medical experience and sense of gain.

## 1. Introduction

Medical complaints are an inevitable event in hospitals.^[1]^ According to the “Measures for the Administration of Complaints from Medical Institution,” medical complaints refer to the activities in which patients report to medical institutions regarding problems in medical service behavior, medical management, and medical quality and safety; patients also provide opinions, suggestions, or appeals to medical institutions to conduct investigation, processing, and result feedback.^[2]^ As a potential, hidden, and useful quality management tool and source channel of valuable information,^[3,4]^ medical complaints have received continuous attention in recent years.^[5–8]^ However, most of the previous studies are based on complaints from a certain region, a certain range, a certain channel or hospitals with a long history as the research object,^[9–12]^ and the medical complaints about newly opened and operated hospitals. Relatively few studies are available, and we still have not paid sufficient attention to medical complaints in actual implementation and have ignored many complaints that may cause serious adverse consequences, which poses huge challenges to hospital management.^[13–16]^

After obtaining the consent of the hospital leaders and the Hospital Quality and Safety Management Committee, this study classified and systematically analyzed the medical complaints of Fujian Provincial Jinshan Hospital since its opening 5 years ago. All methods used in this research follow basic ethical guidelines and regulations.

There is still little research evidence on medical complaints in new hospitals, and further analysis and understanding of this aspect is significant for strengthening hospital management, improving medical quality, and improving healthcare quality. Therefore, this study as a retrospective analysis of medical complaints in newly-built hospitals, we have collected all complaint cases from the 3 main complaint channels in China, and we have improved the classification of complaints and innovated analysis methods on the basis of the actual situation of the hospital. This study explored the occurrence and handling of medical complaints in hospitals and discovered defects in hospital quality and procedures to provide a reference for hospital for optimizing medical procedures, improving medical services, ensuring medical safety, enhancing the patient’s medical experience and protection of the patient’s legal rights and interests, and providing references for newly established and operated hospitals to evade medical complaints and deal with defects related to medical quality and diagnosis and treatment procedures. We hypothesized that new hospitals differ in the content of medical complaints, types of medical complaints, and complaint departments, but do not differ significantly in the timing of complaints, the recipients of complaints, or how complaints are handled.

## 2. Method and data

### 2.1. Hospital selection

This study was conducted in Fujian Provincial Jinshan Hospital. Fujian Provincial Jinshan Hospital is a newly built tertiary first-class general hospital in Fuzhou City. It is located in Jinshan New City, Cangshan District, Fuzhou City, which is the capital of Fujian Province. It is managed and operated by Fujian Provincial Hospital and provides various medical services to approximately 400,000 nearby residents. The hospital has 505 beds, the annual outpatient and emergency visits exceed 500,000, the number of hospitalizations exceeds 10,000, and the number of surgeries exceeds 5000. The workload of outpatients, hospitalizations, and surgeries since the hospital was completed and put into operation (Table 1).

**Table 1:** Visits of Fujian Provincial Jinshan Hospital from 2016 to 2020.

Year	Outpatient and emergency	Hospitalized	Operation
2016	617,500	14,900	4600
2017	671,900	15,200	5600
2018	680,400	16,700	5500
2019	736,500	17,200	5700
2020	698,300	15,200	5100

Jinshan New City is a newly developed area in Fuzhou City. The population of residents is complex, with different ages, backgrounds, education, income, occupations, and medical service needs vary greatly. The collection and analysis of hospital medical complaints are representative and can provide support and assistance for improving the quality of medical services and optimizing of medical service processes.

### 2.2. Data

Our study collected and sorted out all the medical complaints files of the hospital from Jan 2016 to December 2020. These data come from inside and outside the hospital. The complaints received by the hospital’s medical department and the service center reception are mainly in the hospital, and outside the hospital are mainly petition complaints transferred by the administrative department. The actual data included were 1503 medical complaints from January 2016 to December 2020.

Since 2016, Fujian Provincial Hospital has assigned a clerk to manage all the medical complaints, and regularly summarize, sort out and analyze the complaints. These complaints have been collected from 3 main sources: Complaints directly accepted by the Medical Administration Division, which refers to any medical complaints that patients file directly to this department in the hospital; non-medical complaints handled by Reception Center, which are related with service, hospital environment, and treatment process; Complaints passed on by health administrations, which referred to those hospital-related complaints that patients submitted to the health administration departments at all levels. With hierarchical clustering method and by referring to the related researches home and abroad, we sorted and classified these 111 items of complaints into 7main categories and 26 subcategories, based on their contents and nature.

All relevant data that may involve the identity information of the complainant were deleted or hidden to protect the privacy of the complainant.

### 2.3. Statistical analysis

Complaint materials were sorted out and screened, 22 cases of invalid complaints were eliminated (no specific complaint object, no specific complaint content, and no specific complaint requirements), and a total of 1481 valid complaint cases were sorted out.

In accordance with the principle of “multiple complaints of the same patient with the same problem are counted once, a complaint involves multiple content, and multiple complaints are classified separately,” an analysis model was created using the hierarchical clustering method. According to the source of the complaint, the time of the complaint, the content of the complaint, the department of the complaint, the object of the complaint, and the means of resolution, 1481 complaint cases were classified and analyzed, and corresponding discussions were conducted. The medical complaint information was first brought up by 2 independent researchers individually, after which the extracted information was compared to check the match and fit, and finally reviewed by the corresponding author to exclude various selective biases, and all disagreements were discussed and agreed upon by the group.

The collected data were analyzed descriptively and qualitatively to describe the number, type, departmental distribution and source of medical complaints, and the subject of the complaints, and the data were standardized and statistically analyzed by applying IBM SPSS statistical package, version25.0 (SPSS Inc., Chicago, IL). Continuous data were described in terms of percentages, distribution patterns, linear relationships, and development trends, and the analyzed data were verified using one-sided chi-square tests and paired t-tests, with *P* values ≤ .01 considered statistically significant.

## 3. Results

We found through analysis that most of medical complaints came from petitions transferred by the administration department (n = 911, 61.5%). However, the medical complaints from this source showed a downward trend annually, followed by complaints received by the service center (n = 428, 28.9%) that also increased annually, and the complaints received by the medical department remained at a low level and continued to decrease. In the past 5 years, hospital medical complaints had gone through a process of rising-falling-rising. Among them, the most medical complaints were in 2017 (n = 355), and the least was in 2019 (n = 236); medical complaints per 10,000 patients. The incidence was between 3-6 cases, the lowest was in 2019 (3.2 cases/10,000 population), and the highest was in 2017 (5.28 cases/10,000 population) (Table 2).

**Table 2:** Sources of medical complaints of Fujian Provincial Jinshan Hospital from 2016 to 2020.

Year	Medical department	Service center	Administration department	Total
2016	43	55	226	324
2017	37	67	251	355
2018	25	89	201	315
2019	25	102	109	236
2020	12	115	124	251
Total	142	428	911	1481

**Figure 1.**
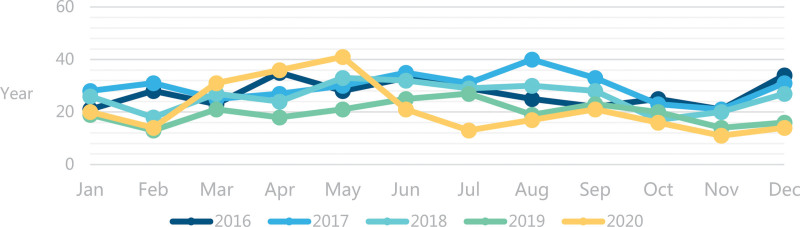
In the figure, the number of complaints in different years is marked with broken lines of different colors. It can be seen from the curve that the number of complaints varies from year to year.

In terms of the time distribution of complaints, May-September was the period of high incidence of medical complaints each year (mean = 25, standard deviation = 7.02). In 5 years, the month with the largest number of complaints was May 2020 (41 cases), followed by August 2017 (40 cases), and the month with the fewest complaints was November 2020 (11 cases) (Fig. 1).

**Figure 2.**
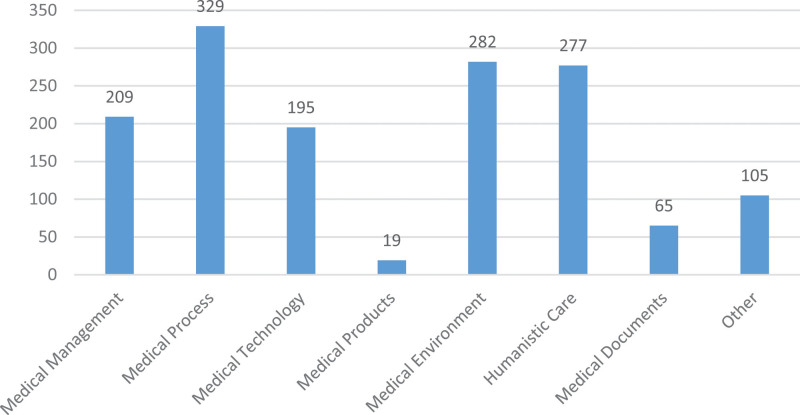
The figure lists the classification and content of hospital medical complaints in the past 5 yr, and the level of the column represents the number of complaints.

Considering the lack of a unified standard for the classification of medical complaints, we referred to the domestic and foreign classification research on medical complaints^[17–21]^ and combined it with the situation of hospital complaints to divide the medical complaints into 7 categories, 26 sub-categories, and 111 specific indicators. After detailed classification, the indicators that do not belong to these 111 indicators were classified under the “other” item. The results showed that our medical complaints in the past 5 years were mainly concentrated in 4 aspects: medical process (n = 329, 22.2%), medical environment (n = 282, 19%), humanistic care (n = 277, 18.7%), and medical management (n = 209, 14.1%). Complaints on medical products (n = 19, 1.3%) and medical documents (n = 65, 4.4%) were relatively few (Fig. 2).

We analyzed 1481 medical complaints by sub-categories and specific indicators. We found that complaints in terms of medical procedures were mainly due to unreasonable medical procedures and cumbersome links (n = 208, 63.2%). In terms of medical environment, the complaints mainly reflected the unreasonable management of parking lots in hospital facilities (n = 127, 45%) and poor diet in hospitals (n = 155, 55%). In the humanistic care, doctor-patient communication was insufficient (n = 169, 61%) and over-medical (n = 67, 24.2%) complaints were common. Complaints in medical management focused on defects in human resource allocation (137, 65.6%), which were mainly due to insufficient outpatient staff arrangements, incomplete department settings, and imperfect medical supplies. Of the 19 complaints on medical products, 16 were related to equipment, which were mainly due to defects in quality, improper use, and failure to keep the labels as required. In the management of medical documents, complaints were mainly because the medical records were not written in time or written/modified in a formal manner (n = 53, 81.5%). In terms of medical technology, the complaints mainly focused on dissatisfaction and nursing (n = 70, 35.9%), medication (n = 54, 27.7%), and surgery/operation (n = 35, 17.9%) (Table 3).

**Table 3:** Contents of medical complaints from Fujian Provincial Jinshan Hospital in the past 5 years.

Category	Subcategory	Specific indicators	n (%)
Medical management (209)	Qualification	2	19 (9.1)
	Resource	3	137 (65.6)
	Rules and regulations	3	53 (25.4)
Medical process (329)	Treatment process	3	208 (63.2)
	Recognition error	3	46 (14)
	Pass on negligence	5	75 (22.8)
Medical technology (195)	Diagnosis	3	12 (6.2)
	Test	2	0 (0)
	Treatment	4	24 (12.3)
	Medication	10	54 (27.7)
	Blood transfusion	5	0 (0)
	Surgery/operation	14	35 (17.9)
	Anesthesia	7	0 (0)
	Nosocomial infection	4	0 (0)
	Nursing	6	70 (35.9)
Medical products (19)	Instrument	7	16 (84.2)
	Pharmacy	5	3 (15.8)
Medical environment (282)	Facilities and equipment	3	127 (45)
	Diet	2	155 (55)
	Security	2	0 (0)
Humanistic care (277)	Doctor-patient communication	4	169 (61)
	Informed consent	4	23 (8.3)
	Privacy infringement	2	18 (6.5)
	Over-medical	2	67 (24.2)
Medical documents (65)	Writing	3	53 (81.5)
	Custody of documents	3	12 (18.5)

**Figure 3.**
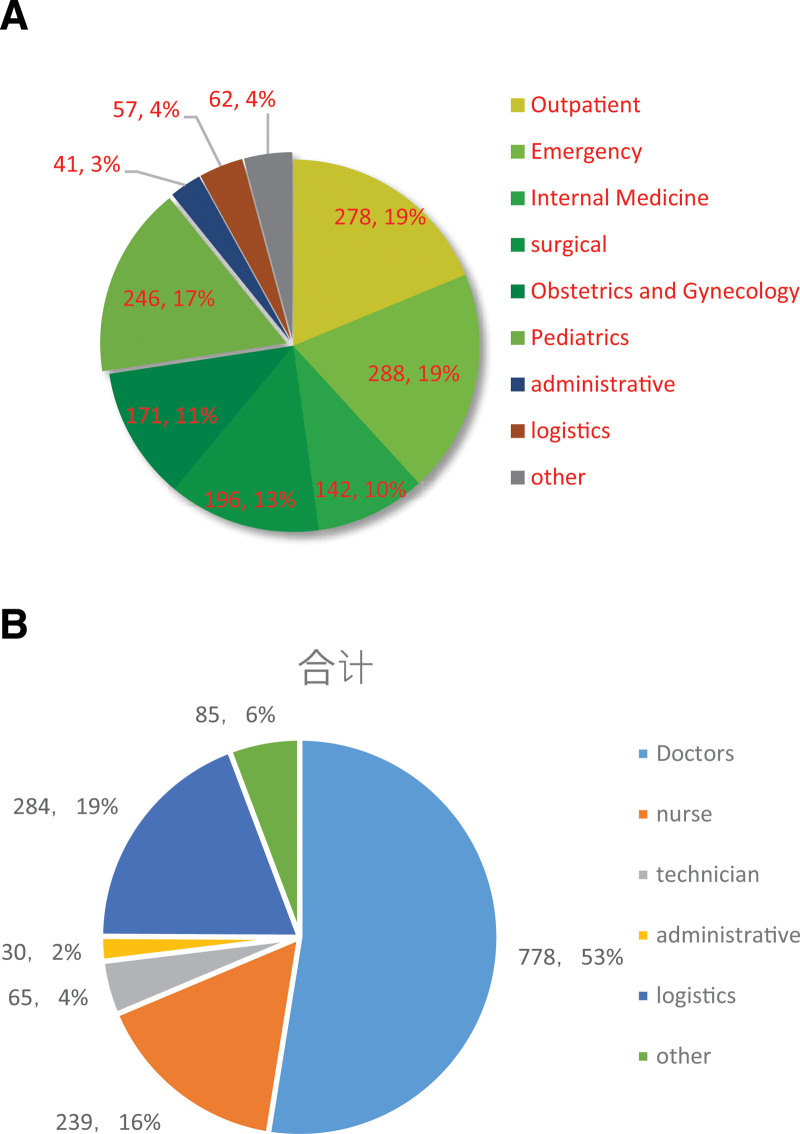
(A) Lists the specific departmental distribution of hospital complaints in the past 5 yr and (B) proportion of different objects of complaints.

The analysis results of complaint departments and subjects showed that the clinical departments encountered more complaints in our hospital. Among them, outpatient, emergency, and pediatric complaints accounted for more than 50% of all medical complaints in the hospital, followed by surgery, women’s obstetrics, and internal medicine. Complaints against logistics support personnel and hospital administrators also increased (Fig. 3A). Among the complaints, doctors were the subject of complaints. Among 1481 medical complaints, 778 (53%) involved doctors, followed by 284 (19%) involving logistics personnel, 239 (16%) involving nurses, 65 (4%) involving technicians, and 30 (2%) involving administrative staff (Fig. 3B).

Most of the 1481 medical complaints made by Fujian Provincial Jinshan Hospital in the past 5 years were properly resolved through letters and calls (n = 1372, 92.6%) and hospital consultations (n = 65, 4.4%), and only a few of them involving serious medical disputes or medical malpractice could only be resolved through third-party mediation (n = 34, 2.3%) and judicial channels (n = 10, 0.7%) (Table 4).

**Table 4:** Ways to resolve medical complaints in Fujian Provincial Jinshan Hospital in the past 5 years.

Year	Letter/phone	Consultation	Mediation	Judicial	Total
2016	297	21	6	0	324
2017	334	14	5	2	355
2018	290	10	12	3	315
2019	210	13	8	5	236
2020	241	7	3	0	251
Total	1372	65	34	10	1481

## 4. Discussion

A certain positive correlation exists between medical complaints and medical business volume.^[22–24]^ However, we find in our research that this correlation is insignificant (*P* = .158). This result does not confirm that the medical business and medical complaints are irrelevant, but it only proves that this relationship can be controlled or even changed by taking practical measures. In our hospital, we have been paying attention to the relationship between medical complaints and business volume since 2016 by focusing on the collection of deficiencies and deficiencies exposed in medical complaints and combining the hospital’s actions to improve medical services, quality and safety enhancement years, and volunteer actions. We will systematically rectify medical defects, improve medical procedures, and optimize medical links. Especially since 2018, we regularly report and display medical complaints in the whole hospital and organize learning and discussions, which helps increase risk awareness of medical staff, improve service quality, enhance service attitudes, and keep harmonious doctor-patient relationship. The growth of the medical business volume can ensure the decline or low increase in medical complaints.

Among the 1481 medical complaints we have obtained, more than half of the medical complaints are from petition complaints transferred by the administrative department (911, 61.5%). Although the hospital has established a relatively standardized and standard complaint acceptance procedure, patients usually do not have a very good understanding of the hospital’s process of receiving and accepting medical complaints and often choose the relatively easy government complaint hotline.^[5,11]^ This situation shows that the hospital’s existing complaint acceptance mechanism is not perfect, and the complaint channels are not smooth and convenient. Therefore, we should further strengthen complaint management, increase publicity, optimize the medical complaint acceptance and reception process, facilitate complaint channels, establish a monitoring and early warning mechanism, and improve the efficiency of complaint acceptance and response in the hospital.^[25]^

After conducting hierarchical clustering of 111 specific indicators in 7 categories and 11 subcategories, we find that the personnel (doctors, nurses, technicians) and clinical departments (outpatient, emergency, pediatrics, obstetrics, and gynecology) are the major groups and departments of medical complaints. The process of treatment and the communication and attitude of medical staff are the main aspects of complaints, which are consistent with the results of other studies.^[5,11,21]^ However, different from previous studies, as a newly built hospital, our focus and concern in complaints about personnel and departments is the lack of medical staff and the incomplete setting of departments. We also have a large number of complaints in terms of hospital facilities, equipment, and diet. This finding reminds our newly opened hospitals to improve the medical humanistic care and communication skills of medical staff and strengthen the communication between doctors and patients. At the same time, we should pay more attention to the provision of medical resources and the improvement of supporting facilities such as logistics support to provide patients with suitable and accessible medical services for creating a warm and comfortable medical environment to meet the needs of patients.^[26,27]^

Although correspondence and telephone calls are our main means to resolve medical complaints, of the 1372 complaints resolved by telephone and correspondence, only 68 cases and less than 5% (68/1372) are replied by formal correspondence, and the rest are all understanding through telephone communication and may be related to traditional Chinese concepts and modern social methods. We are not very good at using formal letters to respond to complaints. We also find through the analysis of the complaint data and resolution methods that the 22 invalid complaints that are excluded and 18% (247/1372) of the 1372 complaints resolved by letter and telephone are simply complaints and venting their dissatisfaction. No specific demands are made in the actual complaint. They will complain as long as they encounter dissatisfaction in any part of the treatment, or if their expected needs are not met and special care is taken. Through communication via telephone, these people are satisfied with the hospital’s explanation. Therefore, for this part of the complaint, only 20% of the time is needed to process the feedback. We should devote more energy to those medical complaints that really warn of defects and loopholes in hospital quality and safety, especially adverse events that can cause medical disputes and medical malpractice; we should also improve the response efficiency and ability of responding to such medical complaints.^[28,29]^

We find after collating and reviewing the data that, out of 99 medical complaints resolved through hospital consultation and third-party mediation, 47% are unsatisfied with the previous telephone response. This part of the patients thought that they are not satisfied with the telephone or in response to complaints in the form of letters; the hospital did not conduct adequate dialogs and investigations, did not provide suitable solutions, and did not pay sufficient attention and maintenance to its rights,^[30]^ which led to the escalation and deterioration of complaints. Therefore, any medical complaints should not be taken lightly at all times. Appropriate and proper ways and means should be adopted to deal with medical complaints to improve the patient’s sense of experience and sense of acquisition.^[31]^

## 5. Research limitation

Our research also has some shortcomings. First, we collect and organize medical complaint data manually, and we lack a standardized and scientific management information system,^[32]^ which may have artificial perception effects on classification, screening, and analysis. Second, when accepting medical complaints, we only register the patient’s gender and do not record other sociodemographic factors, such as age, income, occupation, marital status, and education level; as a result, we could not analysis and discuss the correlation between demographic sociology and medical complaints.^[8]^ Third, Considering the lack of a unified standard for the classification of medical complaint, we have classified 111 specific indicators in 7 categories and 26 sub-categories. However, some complaints cannot be classified, which may miss some important medical complaint information, which is not conducive to the quality improvement and safety of the hospital. Fourth, in actual work, the choice of complaint resolution is random and subjective and depends on the staff’s artificial judgment on the natural and severity of the complaint. In some cases, the hospital chooses to negotiate and resolve the complaint quickly without investigating its cause and background to solve the problem as soon as possible, eliminate the effect, and clam down the matter. This kind of behavior may cause the exclusion and shielding of important information. Thus, the strategies needed for the hospital’s continuous improvement may be difficult to obtain. Fifth, we only included a sample of patients who had complaints about the hospital and did not collect relevant information from other patients, thus lacking comparability to some extent, but we believe that the most valuable to hospitals should be complaints about complaints, not satisfied subjects, and that complaints can significantly and improve hospital services and safety, protect patient rights, and enhance patient experience and access. Finally, our research is carried out in a single hospital, thus the data of complaints has its limitation. But considering the fact that the patients of the hospital covers all ages, the diseases of inpatients admitted in 2020 have reached more than 90% of the national requirements, and the surgical methods carried out in 2020 have covered all surgical types stipulated by the National Health Commission of China, we believe that the data of complaints collected could be representative and our findings could still provide beneficial reference for other hospitals.

## 6. Conclusion

Medical complaints are a valuable source of information, help discover hidden shortcomings and defects in hospitals, and provide strategic help and opportunities for hospital quality improvement and risk management.^[5–9,18,29]^ However, doctors, nurses, or hospital administrators who are afraid, evasive, or even resistant to medical complaints are unwilling to actively collect information related to medical complaints and use medical complaints to improve services and optimize processes. Our research revealed that medical process, medical environment, humanistic care and medical technology are the main source of complaints. Continual attention, optimization and improvement in the above aspects will help increase the quality of medical care, thus further improve patients’ medical experience and enhance satisfaction. We suggest that new hospitals should pay more attention to the services and quality of medical resources and logistical support in the early stage of opening, change their concepts, follow the best practices of patient-centered, improve various medical complaint channels, establish and adopt multiple ways to properly accept and handle medical complaints,^[33]^ build a monitoring and early warning mechanism, enhance the timeliness and feedback efficiency of responding to medical complaints,^[34]^ and strengthen communication, exchanges, and dialogue to maintain and improve patients’ medical experience and sense of gain.

## Acknowledgments

We would like to thank the leaders of Fujian Provincial Hospital and all members of the hospital’s Medical Quality and Safety Management Committee for their strong support and help in this study. Special thanks also go to professor Zhijian Hu of Fujian Medical University for his great help, guidance, and review of the statistics-related knowledge of this study.

## Author contributions

**Data curation:** Jian Jiang.

**Formal analysis:** Jian Jiang.

**Funding acquisition:** Jian Jiang.

**Investigation:** Jian Jiang, Fanqian Huang, Huiting LI.

**Methodology:** Jian Jiang.

**Project administration:** Jian Jiang.

**Resources:** Fanqian Huang, Huiting LI.

**Writing – original draft:** Jian Jiang.

**Writing – review & editing:** Jian Jiang, Fanqian Huang, Huiting LI.
